# Regulation and directed inhibition of ECP production by human neutrophils

**DOI:** 10.3389/fimmu.2022.1015529

**Published:** 2022-11-28

**Authors:** Antonio Vega-Rioja, Pedro Chacón, Lourdes Fernández-Delgado, Bouchra Doukkali, Alberto del Valle Rodríguez, James R. Perkins, Juan A. G. Ranea, Leticia Dominguez-Cereijo, Beatriz María Pérez-Machuca, Ricardo Palacios, David Rodríguez, Javier Monteseirín, David Ribas-Pérez

**Affiliations:** ^1^ UGC de Alergología, Hospital Universitario Virgen Macarena, Sevilla, Spain; ^2^ Departamento de Medicina, Facultad de Medicina, Universidad de Sevilla, Sevilla, Spain; ^3^ Departamento de Biología Molecular y Bioquímica. Facultad de Ciencias, Universidad de Málaga, Málaga, Spain; ^4^ Centro de Investigación Biomédica en Red de Enfermedades Raras (CIBERER), Instituto de Salud Carlos III, Madrid, Spain; ^5^ Instituto de Investigación Biomédica de Málaga-IBIMA, Málaga, Spain; ^6^ Laboratorios Diater, Madrid, Spain; ^7^ Hospital Quirón Sagrado Corazón and Hospital Quirón Infanta-Luisa, Sevilla, Spain; ^8^ Facultad de Odontología, Universidad de Sevilla, Sevilla, Spain

**Keywords:** allergy, asthma, IgE, ECP, allergen, cell signaling, cytokines, immunotherapy

## Abstract

**Background:**

Neutrophils are involved in the pathophysiology of allergic asthma, where the Eosinophil Cationic Protein **(**ECP) is a critical inflammatory mediator. Although ECP production is attributed to eosinophils, we reported that ECP is also present in neutrophils from allergic patients where, in contrast to eosinophils, it is produced in an IgE-dependent manner. Given the key role of ECP in asthma, we investigated the molecular mechanisms involved in ECP production as well as the effects induced by agonists and widely used clinical approaches. We also analyzed the correlation between ECP production and lung function.

**Methods:**

Neutrophils from allergic asthmatic patients were challenged with allergens, alone or in combination with cytokines, in the presence of cell-signaling inhibitors and clinical drugs. We analyzed ECP levels by ELISA and confocal microscopy. Lung function was assessed by spirometry.

**Results:**

IgE-mediated ECP release is dependent on phosphoinositide 3-kinase, the extracellular signal-regulated kinase (ERK1/2) and the production of reactive oxygen species by NADPH-oxidase. Calcineurin phosphatase and the transcription factor NFAT are also involved. ECP release is enhanced by the cytokines interleukin (IL)-5 and granulocyte macrophage-colony stimulating factor, and inhibited by interferon-γ, IL-10, clinical drugs (formoterol, tiotropium and budesonide) and allergen-specific IT. We also found an inverse correlation between asthma severity and ECP levels.

**Conclusions:**

Our results suggest the molecular pathways involved in ECP production and potential therapeutic targets. We also provide a new method to evaluate disease severity in asthmatic patients based on the quantification of *in vitro* ECP production by peripheral neutrophils.

## Introduction

Eosinophil Cationic Protein (ECP) is a highly basic protein with cytotoxic and non-cytotoxic properties that displays multiple functions, including RNAse activity. It is involved in host defense against parasites, bacteria and viruses, and regulates immune and epithelial cell function, complement, coagulation and fibrinolysis (1). ECP was traditionally considered to be produced only by eosinophils (2). These cells play a predominant role in inflammatory disorders such as asthma, a chronic airway disease affecting more than 300 million people, causing 1 of every 250 deaths worldwide (3). Quantification of eosinophil proteins, including ECP, is commonly used as a tool to assess allergic asthma (4). For instance, high levels of ECP in sputum or bronchoalveolar lavage fluid from asthmatic patients is a clinical marker of eosinophilic infiltration into the airways (2, 5). Immunocytochemistry analysis of these fluids shows that the number of ECP^+^ cells is higher than the number of eosinophils, suggesting that other cell types might also be involved in ECP release in the airways (6). ECP has also been detected in other leukocytes, such as neutrophils (7, 8), indicating that they might be one of these additional sources.

Neutrophils are the first cells to reach the inflammation foci in the asthmatic airways, where they perform inflammatory functions (9). Patients with severe and/or persistent asthma and chronic airflow obstruction display a neutrophil burden in lung tissues (10). In addition, an increased neutrophil count in sputum is associated with acute exacerbations of asthma and lung dysfunction (11).

The expression of IgE receptors has been reported in neutrophils (12–14). We previously showed that some neutrophil functions are activated in response to allergens (Ags) through an IgE-dependent mechanism, including the production of inflammatory mediators, respiratory burst and degranulation (15). Neutrophils from allergic patients, but not eosinophils, produce and release ECP after the challenge of surface-bound IgE with anti-IgE antibodies a-(α-IgE), or Ags to which they are sensitized (16). Given the importance of ECP in allergic asthma pathophysiology, it is important to identify the cellular sources and the molecular mechanisms involved in its production. Our results will be useful to assay clinical approaches for allergic asthma treatment based on target inhibition. This work also provides insights to evaluate to what extent current treatments have a direct effect on neutrophil ECP production.

## Material and methods

### Ethics statement

The Hospital Universitario Virgen Macarena ethics committee approved the study and each sample donor gave written informed consent.

### Chemicals and reagents

The Ags, available as commercial extracts, included D_1_ (*Dermatophagoides pteronyssinus*), G_3_ (*Dactylis glomerata*), T_9_ (*Olea europaea)*, M_6_ *(Alternaria alternata)* and W_6_ (*Artemisia vulgaris*) and were purchased from Diater, (Madrid, Spain). Wortmannin, PD098059, SB203580, 4-hydroxy-3-methoxyaceto-phenone (HMAP), 4-(2-aminoethyl) benzenesulfonyl fluoride (AEBSF), cyclosporin A (CsA), cell permeable NFAT inhibitor (VIVIT), tiotropium bromide, budesonide, formoterol, were from Sigma-Aldrich Co (Madrid, Spain). IL-4, IL-5, GM-CSF, IFN-γ, IL-10 were from Preprotech (Rocky Hill, NJ, USA). Ficoll-hypaque, phosphate-buffered saline, RPMI 1640, fetal bovine serum, penicillin/streptomycin and goat anti-human IgE (α-IgE) were purchased from Thermo-Fisher Invitrogen (San Diego, CA, USA). All cultured reagents had endotoxin levels of ≤ 0.01 ng/ml, as tested by the *Limulus* lysate assay (Coatest, Chromogenix, Mölndal, Sweden).

### Patients and controls

The study included three groups of adult donors: atopic patients with bronchial asthma (17) with no Ag-specific immunotherapy treatment (non-IT), atopic patients with bronchial asthma treated with Ag-specific immunotherapy (IT), and healthy donors (HD) (see **Table 1**). The group of asthmatic patients gave positive skin-prick test (SPT) results (Diater) and serum specific-IgE (HYTEC 288, Hycor Biomedicals, Germany) levels ≥50 KU/l to at least one of the inhalant Ags included in the routine testing battery (house-dust mites, pollens, molds and animal danders). Those Ags to which the patients had specific IgE levels ≥50 KU/l were used for the challenge in the experiments. The non-IT group did not receive specific hyposensitization and did not experience episodes of respiratory infections for the last 4 weeks before blood extraction. The IT group received Ag-specific IT (Diater) for the previous three years and continued to receive a maintenance dose ofthe highest dose of the extract. Allergic patients did not take any inhaled bronchodilators within 8 h before cell isolation and the *in vitro* cellular challenge, oral bronchodilators for 24 h or antihistamines, oral corticosteroids, disodium cromoglycate, or nedocromil sodium in the previous week. The healthy group had no history of allergy or bronchial symptoms, and gave negative skin-prick tests and had specific-IgE to the battery of inhalant Ags. None of the participants in this study suffered infection by SARS-CoV-2 in the month previous to the blood extraction.

**Table 1:** Demographic characteristics of the study groups.

Parameter	HD(n = 10)	Non-IT AP(n = 30)	IT- AP(n = 10)
Age*	47.2 ± 5.2	45.1 ± 3.7	39.6 ± 10.1
Gender (♂/♀)	5/5	17/13	3/7
Caucasian (%)	100	100	100
IgE (KU/L)*	8.9 ± 3.7	410.8 ± 25.7	315 ± 21.9

*Mean ± S.E.M; HD, Healthy donors; AP, allergic patients.

### Cell isolation and culture

Highly purified human peripheral blood neutrophils were isolated using the neutrophil isolation kit (Miltenyi Biotec S.L., Madrid, Spain) following the manufacturer’s instructions. The purity of neutrophils was on average >99% (16). Cells (10^6^ cells/300 μl) were cultured in RPMI 1640 medium supplemented with 10% (v/v) FBS, 2 mM L-glutamine, 100 U/ml penicillim, and 100 μg/ml streptomycin and maintained in an atmosphere of 95% O_2_ and 5% CO_2_. For the stimulation treatments, neutrophils were incubated with 10 μg/ml Ags or α-IgE antibodies at 37°C for the indicated times. Wortmannin, PD098059, SB203580, HMAP, AEBSF, CsA, VIVIT, IL-4, IL-5, IFN-γ, IL-10, GM-CSF, tiotropium, budesonide, and formoterol were added 1 h prior to stimulation and they were previously tested for the optimal concentration without affecting cell viability. In this case, cell health was assayed using the AlamarBlue kit (Thermo-Fisher) according to the manufacturer’s instructions. This kit quantifies the natural reducing power of living cells to convert resazurin to fluorescent resorufin.

### ECP release

ECP released was measured in the culture supernatants by ELISA (CAP system immunoassay; Phadia-Thermo scientific) according to the manufacturer’s instructions.

### Lung function

FEV1 was measured using a spirometer (Vitalograph, Buckingham, UK). The best value of three maneuvers was expressed as the percentage of the predicted value. The entire procedure was based on the guidelines of the American Thoracic Society of Standardization (18).

### Sputum induction, processing, immunocytochemistry and confocal microscopy analysis

Sputum was induced and processed as previously described (19). Briefly, patients inhaled 4.5% hypertonic saline solution at room temperature which was nebulized by an ultrasonic nebulizer (Ultraair NE-U17, Omron Corporation, Japan) at maximal saline output for a 20 min period. Sputum plugs were isolated from the sample and resuspended in PBS supplemented with 100 U/ml penicillin, and 100 μg/ml streptomycin. For ECP levels quantification, samples were directly vortexed and centrifuged (4,000 x g, 10 min, 4°C), and the resulting supernatants were frozen at -80° C until ELISA determination. For cell culture, PBS resuspended plugs were diluted 1/10 in 0.2% dithiothreitol and mechanically mixed for 30 min to disperse the cells. Samples were filtered through a 50 μm strainer and centrifuged for 10 min at 4°C. Processed sputum pellets were resuspended in complete RMPI medium, cells plated on glass coverslips coated with poly-L-lysine and fibrinogen and cultured in the presence or absence of stimulus for 18h. After culture, coverslips were fixed for 30 min with 4% paraformaldehyde in PBS, permeabilized for 15 min at room temperature with 0.5% Triton X-100 in PBS and immediately blocked for 1 h with 1% bovine serum albumin serum in PBT (0.1% Triton X-100 in PBS). After blocking, mouse fluorescein isothiocyanate-conjugated α-human RNase3/ECP mAb (LSBio, Seatle, WA, USA) and mouse phycoerythrin-conjugated α-human Myeloperoxidase mAb (PE-MPO, Beckman-Coulter) diluted in blocking solution at 1:100 final concentration were added and incubated at room temperature for 2h. After washing with PBT, coverslips were mounted onto glass slides with DAPI-containing mounting medium. Cells were imaged using a Stellaris 5 laser scanning confocal microscope from Leica. For 2D, images were obtained using a 20X objective. For 3D, serial optical sections (z-stacks) were obtained using a 63X objective and deconvoluted using ImageJ/Fiji.

### Statistical analysis

All statistical analyses were performed using GraphPad Prism 4.00 for Windows (GraphPad Software, San Diego, CA, USA) and R-4.2.1. Multi-group analysis was performed using two-way ANOVA, followed by *post-hoc* Tukey’s honest significance tests. Correlation was measured using the Pearson correlation coefficient. Data are expressed as mean ± SEM. A value of p<0.05 was considered significant.

## Results

### Cell-signaling pathways involved in the Ags/α-IgE-dependent ECP release

In previous work, we reported that the crosslinking between Ags/α-IgE and IgE molecules bound to surface IgE receptors (Galectin-3> FcεRI) induces the synthesis and release of ECP by human neutrophils from allergic asthmatic patients (16). This prompted us to study the molecular mechanisms underlying this process by treating cells with specific inhibitors of key signaling pathways in neutrophils and measuring their effects on ECP release after treatment with and without Ags or a-(α-IgE).

During the first steps of neutrophil activation there is an increase in phosphoinositides through PI3K activation (20), as well as Mitogen-Activated Protein Kinases (MAPKs) (p38 and extracellular signal-regulated kinase 1/2 (ERK) activation) (21). PI3K and MAPKs regulate the functional assembly of NADPH oxidase (NOX-2) (22), an enzyme producing Radical Oxygen Species (ROS), second messengers involved in the priming and degranulation of neutrophils (23). We have previously shown that PI3K, MAPKs and NOX-2 pathways are activated in neutrophils from allergic patients by Ags/α-IgE (24, 25).

In addition, we have shown that the calcineurin phosphatase (CN)/NFAT transcription factor signaling pathway, a key player in neutrophil activation during the immune responses (26), is also activated in an IgE-dependent manner (27).

In this context, we aimed to explore the specific role of all these molecular intermediates in ECP production. To this end, we tested the effects of the following protein-specific inhibitors: wortmannin (PI3K inhibitor (28)), PD098059 (MEK inhibitor, the upstream kinase of ERK 1/2 (29)), and SB20358 (p38 MAPK inhibitor (30)), HMAP and AEBSF (NOX-2 inhibitors (31)), CsA (CN activity inhibitor (32)) and VIVIT (peptide blocking the interaction CN-NFAT, required for NFAT nuclear translocation (33)).

**Figure 1:**
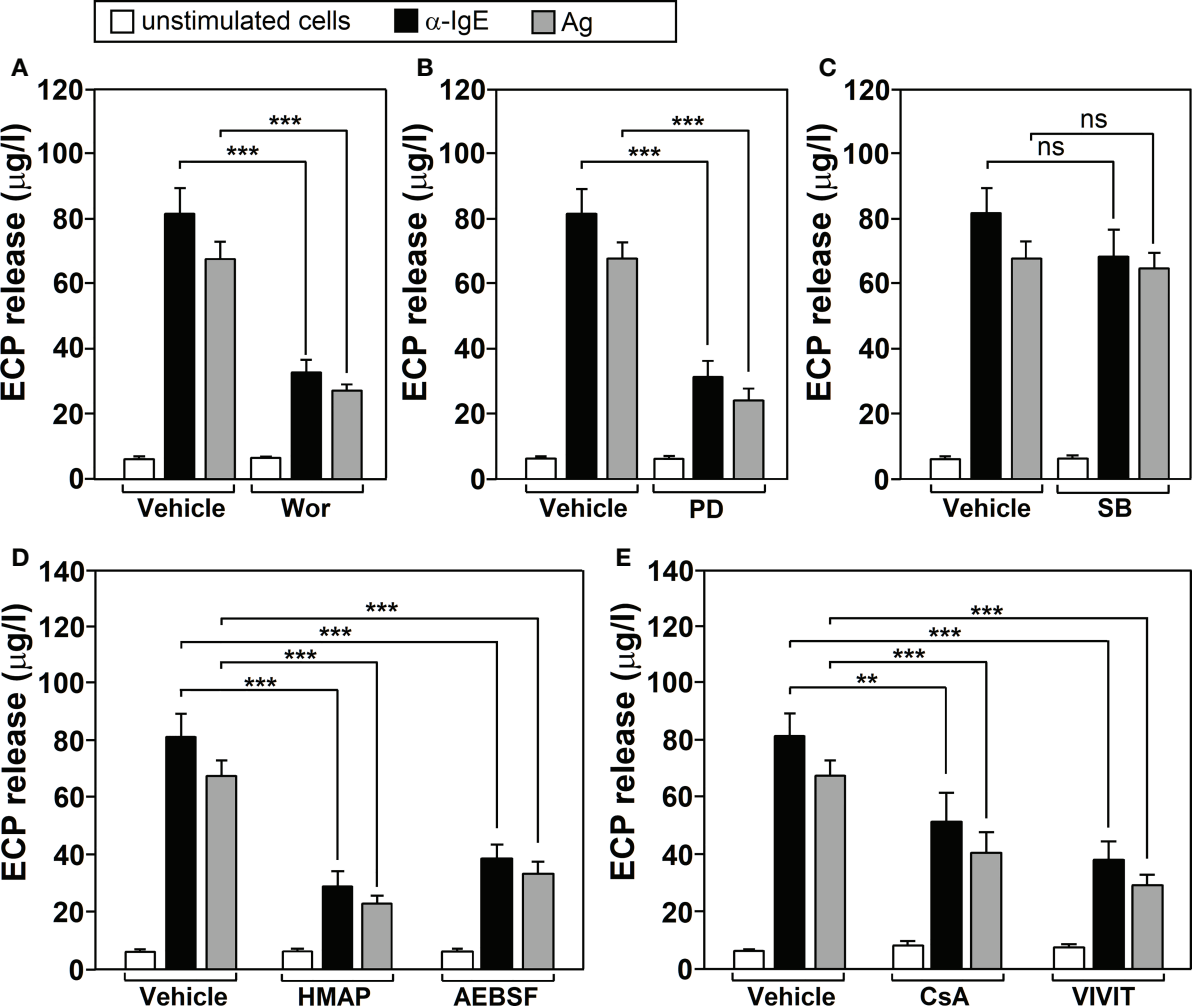
Cell‐signaling pathways involved in Ags-dependent ECP release by human neutrophils. Neutrophils from allergic asthmatic patients (n=5) werepre‐incubated for 1 h with vehicle or with 100 nM wortmannin (Wor, PI3K inhibitor) **(A)**, 10 mM PD098059 (PD, MEK inhibitor) **(B)**, 10 mM SB203580 (SB, p38 MAPK inhibitor) **(C)**, 500 μM HMAP/AEBSF (NOX-2 inhibitors) **(D)** or 1μg/ml CsA (calcineurin inhibitor)/15 μg/ml VIVIT peptide (NFAT inhibitor) **(E)**. They were then left untreated (unstimulated cells) or challenged with a-IgE (10 μg/ml) or with an Ag to which the patients were sensitized (10 μg/ml) for 18 h. The levels of ECP release were determined as indicated in M&M section. Data are the mean ± SEM of five separate experiments, each measurement performed in triplicate. Comparisons performed using Post-hoc Tukey’s HSD test. Comparisons between groups denoted by brackets. ***p< 0.001; **p<0.01. n.s, non-significant. Ags used for the challenge were: T9 (n=3) and G3 (n=2).

Inhibitor treatment (F=24.32, df=7, p<2.2x10^-16^) and stimulation with Ags/α-IgE (F=327.10, df=2, p<2.2x10^-16^) both significantly affected neutrophil ECP release and there was a significant interaction between these factors (F=6.32, df=14, 6.422x10^-09^). Tukey’s *post-hoc* tests showed that wortmannin (**Figure 1A**), PD098059 (**Figure 1B**), HMAP and AEBSF (**Figure 1D**) and CsA/VIVIT (**Figure 1E**) significantly inhibited ECP release induced by Ags/α-IgE. Conversely, SB203580 did not have any significant effect (**Figure 1C**) (p=0.99 for Ags treatment and p=0.77 for α-IgE treatment). For all molecules tested, no changes in ECP release could be detected following treatment in the absence of stimulation.

### Regulation of neutrophil ECP production by cytokines

Neutrophil ECP production is modulated by agonists such as Platelet Activating Factor (PAF) (16). To study whether other cytokines could also modulate ECP release by human neutrophils, we examined the effect of two groups: IL-4, IL-5 and GM-CSF, that promote allergic inflammation (34, 35), and IFN-γ (36) and IL-10 (37), that inhibit this process.

Neutrophils from allergic patients were preincubated with these cytokines 1h prior the addition of Ags/α-IgE. We then quantified ECP production in the culture supernatant. IL-4 did not modify the basal nor the IgE-dependent ECP production. The same dose of IL-5 did not have an effect on its own, but it significantly enhanced the Ags/α-IgE stimulating effect. Interestingly, GM-CSF displayed a robust response in Ags/α-IgE-treated cells, and was the only cytokine producing significant ECP release in unstimulated cells. On the other hand, IFN-γ and IL-10 significantly reduced ECP release in both stimulated and unstimulated cells (**Table 2**).

**Table 2:** Effect of cytokines on neutrophil ECP release.

Experimental condition	ECP (µg/l)	*P* value
Unstimulated cells (vehicle)	6.3 ± 0.4	
α-IgE	82.1 ± 12.3	p<0.001 *** _(1)_
Ags	67.7 ± 10.5	p<0.001 *** _(1)_
IL-4	5.8 ± 0.5	p=0.483 n.s _(1)_
α-IgE + IL-4	79.9 ± 9.5	p=0.180 n.s _(2)_
Ags + IL-4	56.6 ± 7.1	p=0.067 n.s _(3)_
IL-5	9 ± 0.7	p=0.140 n.s _(1)_
α-IgE+ IL-5	174.4 ± 24.4	p=0.004 ** _(2)_
Ags + IL-5	122.6 ± 10.1	p=0.003 ** _(3)_
GM-CSF	28.5 ± 2.9	p<0.001 *** _(1)_
α-IgE + GM-CSF	277.4 ± 29.1	p<0.001 *** _(2)_
Ags + GM-CSF	190.7 ± 36.0	p=0.002 ** _(3)_
IFN-γ	1.3 ± 0.6	p=0.045 * _(1)_
α-IgE + IFN-γ	33.5 ± 5.2	p=0.004 ** _(2)_
Ags + IFN-γ	39.2 ± 5.3	p=0.003 ** _(3)_
IL-10	2.4 ± 0.4	p=0.023 * _(1)_
α-IgE+ IL-10	32.9 ± 5.0	p=0.004 ** _(2)_
Ags + IL-10	28.4 ± 4.6	p=0.002 ** _(3)_

Cells from allergic asthmatic patients (n=5) were left untreated (vehicle) or were treated with IL-4 (100 U/ml), IL-5 (100 U/ml), GM-CSF (50 U/ml), IFN-γ (100 U/ml) and IL-10 (100 U/ml) in the presence or absence of Ags to which they were sensitized (10 µg/ml) or α-IgE (10 μg/ml) for 18 h. The levels of ECP released were determined as indicated in M&M section. Data are the mean ± SEM of five separate experiments in which each measurement was performed in triplicate. Comparisons made using Student’s t test (two tailed). (1): p vs unstimulated cells; (2): p vs α-IgE treated cells; (3) p vs Ags-treated cells; *p < 0.05; **p < 0.01; ***p < 0.001. IL-4: interleukin-4; IL-5: interleukin-5; IL-10: interleukin-10; GM-CSF: Granulocyte Macrophage-Colony Stimulating Factor; IFN-γ: Interferon-γ. n.s, non-significant.

### The effects of therapeutic approaches on IgE-dependent ECP release

Glucorticosteroids (GC) (38), bronchodilators (Long-Acting Beta_2_-Agonists, LABAs) (39), and Long-Acting Muscarinic Antagonists, LAMAs) (40) are effective drugs for preventing allergic symptoms. We investigated their possible modulating effect on IgE-dependent ECP release. We tested the action of budesonide (a GC), formoterol and tiotropium (a LABA and a LAMA, respectively) by adding them to the culture of neutrophils from allergic patients (prior to Ags/α-IgE antibodies stimulation) and quantified ECP release.

**Figure 2:**
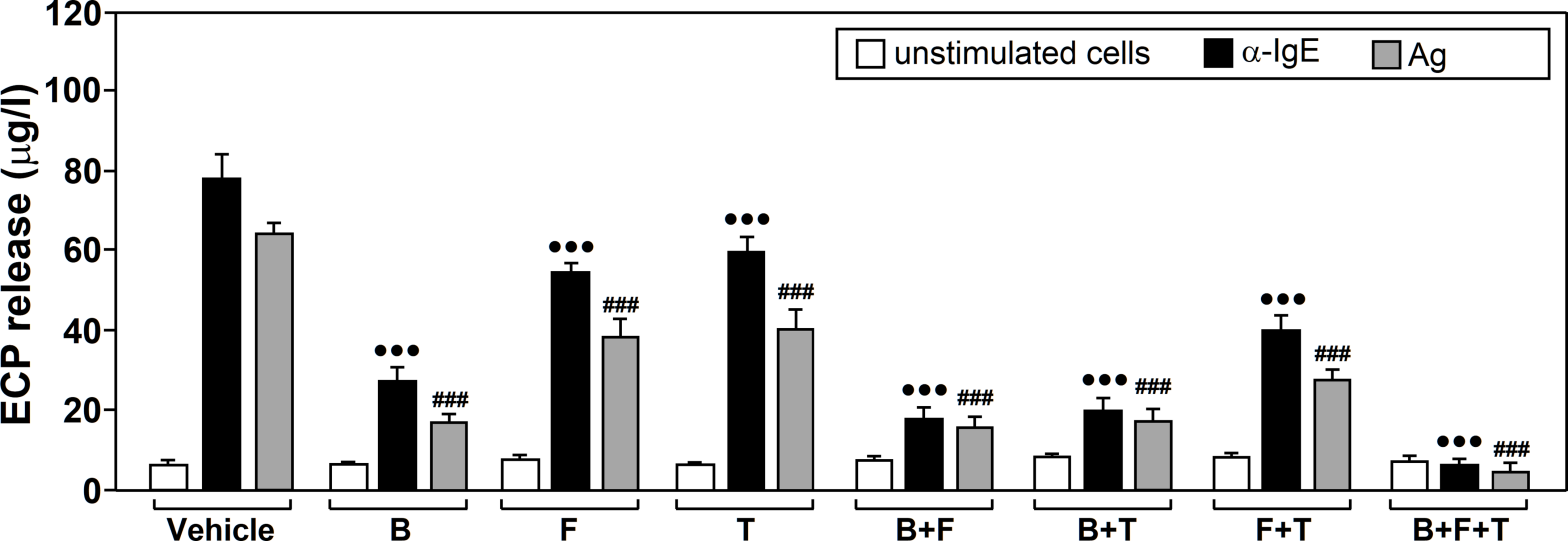
The effects of glucocorticoids, Long-Acting β_2_-Agonists (LABAs) and Long-Acting Muscarinic Antagonists (LAMAson Ags/α-IgE-dependent ECP release. Neutrophils from allergic asthmatic patients (n=7) were pre‐incubated for 1 h with vehicle or treated with 10 µM budesonide (B, glucocorticoid), formoterol (F, LABA), tiotropium (T, LAMA), budesonide plus formoterol (B+F), budesonide plus tiotropium (B+T), formoterol plus tiotropium (F+T), or budesonide plus formoterol plus tiotropium (B+F+T). They were then left untreated (unstimulated cells) or challenged with α-IgE (10 µg/ml) or with an Ag to which patients were sensitized (10 µg/ml) for 18 h. ECP release was measured in the culture supernatants by ELISA. Data are expressed as the mean ± SEM from seven separate experiments, each measurement performed in triplicate. Comparisons were made with the vehicle treated cells. Comparisons performed using *Post-hoc* Tukey’s HSD test: ^•••^p< 0.001 drug-treated *vs* vehicle-treated cells, both stimulated with α-IgE. ^###^p < 0.001 drug-treated *vs* vehicle-treated cells, both stimulated with Ags. Ags used for the challenge were: T_9_ (n=3), G_3_ (n=2) and D_1_ (n=2).

Our experiments revealed that drug treatment (F=101.45, df=7, p<2.2x10^-16^) and stimulation (F=327.10, df=2, p<2.2x10^-16^) both significantly affected neutrophil ECP release and there was a significant interaction between these factors (F=28.58, df=14, p<2.2x10^-16^). Tukey’s *post-hoc* tests showed that all three drugs, alone or in combination, led to a significant reduction in ECP release, compared to untreated cells, for both Ags and α-IgE stimulation. Considering the drugs alone, Budesonide led to the largest change, followed by formoterol and tiotropium (**Figure 2**).

Current medical guidelines recommend the use of a triple therapy combining GC, LABAs and LAMAs for the non-controlled asthma (e.g. GINA) (41). Thus, we perfomed the same experiments using cocktails of these drugs.

The combination of formoterol and tiotropium enhanced the inhibitory effect that they produced alone. This inhibition was more significant when formoterol or tiotropium were combined with budesonide. The mix with all three drugs showed the largest decrease in ECP release (**Figure 2**). No changes were detected for any of the treatments in absence of stimulation.

### The effect of Ag-specific IT on Ags/α-IgE-, IL-5-, and GM-CSF-dependent ECP release by human neutrophils

**Figure 3:**
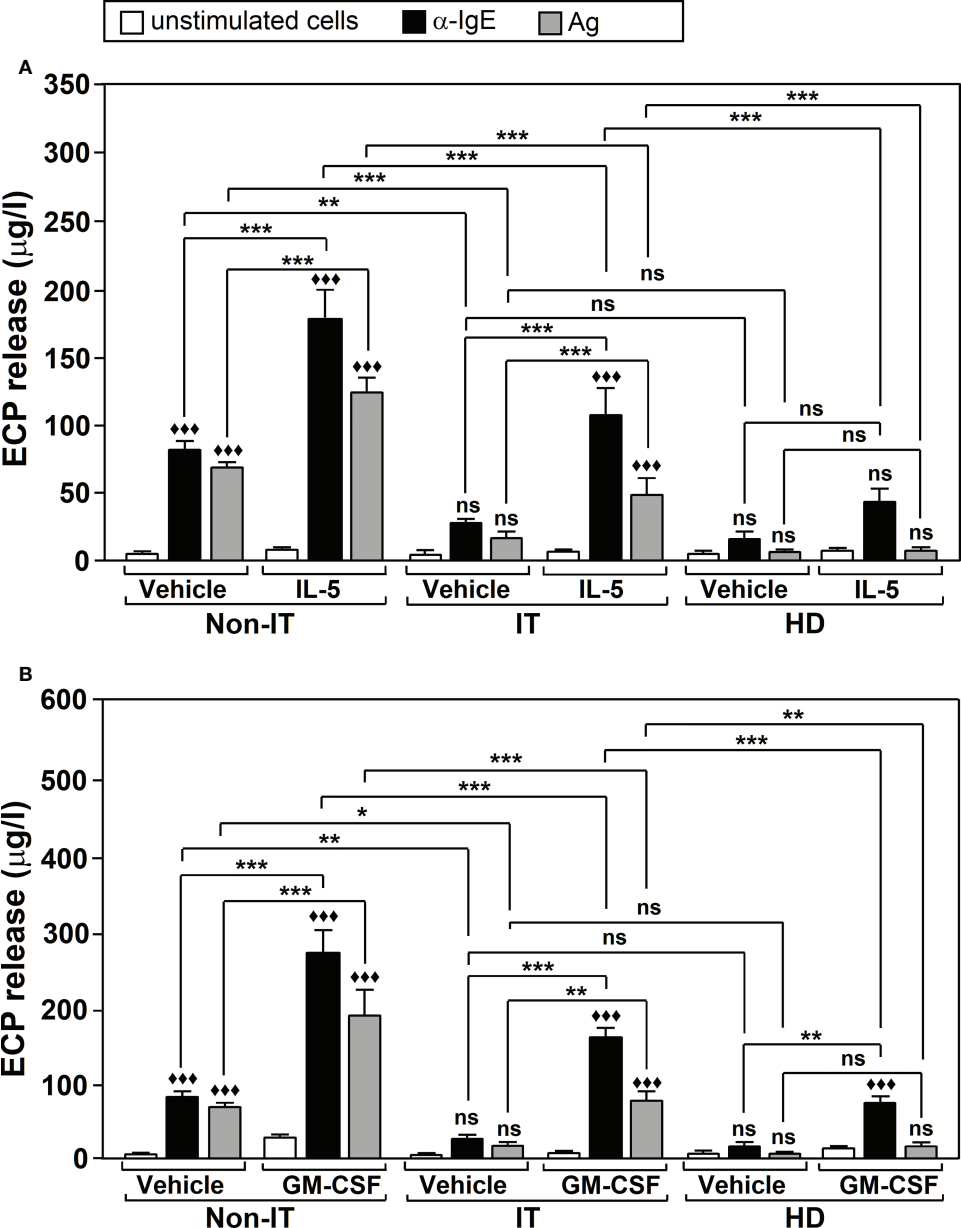
The effect of Ags-specific IT on Ags/α-IgE-, IL-5-, and GM-CSF-dependent ECP release. Neutrophils were purified from non-IT-treated allergic patients (Non-IT; n = 10), IT-treated allergic patients (IT; n=10) or healthy donors (HD; n=10). Cells were incubated with vehicle or with 100 U/ml IL-5 **(A)** or 50 U/ml GM-CSF **(B)** and then left untreated (unstimulated cells) or challenged with Ags (10 µg/ml) or α-IgE (10 µg/ml) for 18 h. ECP release was measured in the culture supernatants by ELISA. Data are the mean ± SEM from 10 separate experiments, each measurement performed in triplicate. Comparisons performed using *Post-hoc* Tukey’s HSD test. Comparisons within treatment groups (GM-CSF/IL-5/Vehicle vs. unstimulated) are denoted by: ^♦♦♦^p < 0.001; ns: non-significant. Comparisons between treatment groups are denoted by brackets. ***p < 0.001, **p < 0.005, * p<0.05. ns, non-significant. Ags used for the challenge were: T_9_ (n=4 for IT, n=4 for non IT, n=4 for HD donors), G_3_ (n=3 for IT, n=3 for non IT, n=3 for HD donors) and D_1_ (n=3 for IT, n=3 for non IT, n=3 for HD donors). IL-5: interleukin-5; GM-CSF, Granulocyte Macrophage-Colony Stimulating Factor.

Ag-specific IT provides long-term reduction in both allergic symptoms and disease progression (42). This improvement correlates with a decline in inflammatory parameters such as ECP, for which nasal or sputum levels decrease in allergic patients after IT treatment (43, 44). This compelled us to analyze whether IT had any effect on Ags/α-IgE-induced ECP production, as well as on the modulating action of IL-5 and GM-CSF. We evaluated ECP release by neutrophils in three groups of subjects: non-IT treated allergic patients, IT-treated allergic patients and healthy donors. Measurements were performed in the culture supernatants of untreated or Ags/α-IgE treated cells, in the presence or absence of either IL-5 or GM-CSF. We found that IL-5/GM-CSF treatment (F=60.88, df=8, p<2.2x10^-16^) and Ags/α-IgE stimulation (F=175.79, df=2, p<2.2x10^-16^) significantly affected ECP release and there was an interaction between factors (F=13.80, df=16, p<2.2x10^-16^). Further analysis using Tukey’s *post-hoc* tests showed that the group of IT-treated allergic patients experienced a significant reduction in ECP release in the presence of Ags/α-IgE, compared with the non-IT treated patients (**Figures 3A, B**). This reduction was ~70% (For α-IgE treatment: 29.80 ± 1.94 μg/l in IT-treated allergic patients *vs* 82.90 ± 5.39 μg/l in non-IT-treated allergic patients; For Ags treatment: 18.60 ± 3.15 μg/l in IT-treated allergic patients *vs* 69.48 ± 3.11 μg/l in non-IT-treated allergic patients). Although there was no significant difference in ECP release between IT-treated patients and healthy donors, values were higher in the first group with respect to the second (for α-IgE treatment: 29.80 ± 1.94 μg/l in IT-allergic patients *vs* 17.40 ± 4.23 μg/l in healthy donors; For Ags treatment: 18.60 ± 3.15 μg/l in IT-allergic patients *vs* 7.45 ± 0.74 μg/l in healthy donors). Note that in healthy donors, neither Ags nor α-IgE led to significant differences in ECP release compared with unstimulated cells, although α-IgE treatment did suggest a possible increase.

Regarding IL-5 and GM-CSF, we found that both cytokines induced a significant ~2-3 fold increase in Ags/α-IgE-induced ECP production for both non-IT and IT-treated allergic patients. However, ECP release was significantly lower (p<0.001) in patients who received IT treatment compared to patients who did not (**Figures 3A, B**).

In healthy donors GM-CSF led to a significant increase in ECP release after α-IgE stimulation (75.87 ± 5.11 μg/l in cells treated with GM-CSF + α-IgE *vs* 17.40 ± 4.23 μg/l in cells treated only with α-IgE, p < 0.005) (**Figure 3B**). For IL-5 there was also an increase but this was not significant (44.82 ± 7.47 μg/l in cells treated with IL-5 + α-IgE *vs* 17.40 ± 4.23 μg/l in cells treated only with α-IgE, p = 0.151) (**Figure 3A**). ECP release was significantly lower in healthy donors after stimulation than that in IT-treated allergic patients (p < 0.001) (**Figure 3**).

### ECP release and lung function

**Figure 4:**
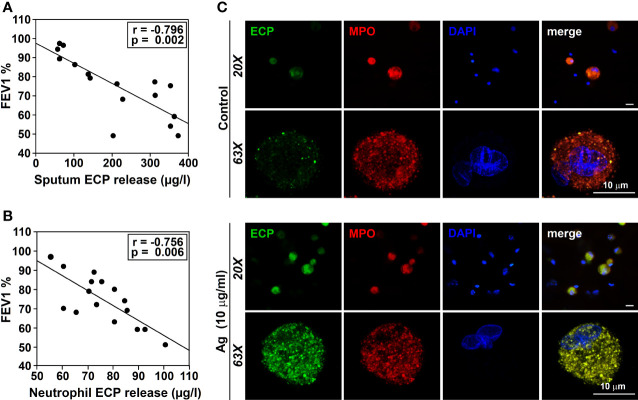
Relationship between ECP release and lung function. ECP production by neutrophils from induced-sputum. **(A)** FEV1 was measured in the exhaled air of allergic asthmatic patients (n= 4 sensitized to T_9_, n=3 sensitized to G_3_, n=3 sensitized to D_1_, n=3 sensitized to W_6_ and N=3 sensitized to M_6_) as described in M&M. ECP release was measured in the induced-sputum from these patients by ELISA. The amount of ECP released in the sputum was evaluated vs FEV1. A significant inverse correlation was observed (r=-0.796, p=0.002). **(B)** FEV1 was measured in the exhaled air of allergic asthmatic patients (n=16) as described in M&M. Blood isolated neutrophils from these patients were treated with 10 µg/ml of Ags to which the allergic patients were sensitized for 18 h. ECP release was measured in the culture supernatants. The amount of ECP released *in vitro* by neutrophils from these patients was evaluated vs FEV1. A significant inverse correlation was observed (r=-0.769, p=0.005). Ags used in **(A)** and **(B)** for the challenge were: T_9_ (n=4), G_3_ (n=3), D_1_ (n=3), W_6_ (n=3), M_6_ (n=3). **(C)** Sputum cells from an allergic patient were isolated as described in M&M and cultured for 18 in the presence or absence of Ags (T_9_, 10 µg/ml) to which the patient was sensitized. Cells were then stained with anti-ECP (green), anti-MPO Ab (red) and DAPI nuclear counterstain (blue). Note that the residual ECP signal detected in resting cells (control) was increased in T_9_-stimulated neutrophils from an allergically-sensitized patient, co-locating with the MPO signal (yellow in the merge). The images are representative of samples obtained from 4 other allergic asthmatics patients.

The inflammatory process of the airways, typical of asthma pathophysiology, induces tissue remodeling resulting in abnormal lung function (45). In asthmatic patients induced-sputum, ECP levels are inversely correlated with lung function (46), and increase after Ags nasal challenge (47). ECP levels are used as a clinical marker of the disease, assuming that they reflect eosinophilic inflammation in the airways (2). However, the numbers of ECP^+^ cells are not always correlated with eosinophil counts (6), suggesting the presence of other sources of ECP. Thus, we conducted our next set of experiments to study the relationship between ECP and lung function. We selected a group of allergic asthmatic patients, and collected induced-sputum to measure ECP levels in relation to the lung function (measured as FEV1). Additionally, we analyzed *in vitro* Ags-induced ECP production by peripheral blood neutrophils isolated from the same donors. In agreement with previous data (46), we observed a significant inverse correlation between induced-sputum ECP levels and lung function (**Figure 4A**). Strikingly, ECP levels released by blood neutrophils versus the corresponding FEV1, also showed a significant inverse correlation (**Figure 4B**). These results indicate that *in vitro* peripheral neutrophil ECP is a potential marker of airway inflammation/asthma severity.

Sputum contains a mixed-cell population consisting of squamous epithelial cells and a small fraction of leukocytes (neutrophils, eosinophils, macrophages/monocytes and lymphocytes). ECP has been immunohistochemically detected not only in eosinophil granulocytes, but also in neutrophils (6). We could thus assume that neutrophils in the airways will behave similarly to neutrophils in the blood, releasing the same mediators in response to the same stimuli. However, neutrophils from different tissues (airways *vs* blood) can behave differently (48). Therefore, we tested whether neutrophils from induced-sputum contribute to IgE-dependent ECP production, similar to peripheral blood. To this end, we cultured leukocyte-enriched cell populations from induced sputum of asthmatic patients in the presence of the Ags to which they were sensitized. We analyzed ECP expression by confocal microscopy. A residual green fluorescence was detected in resting sputum neutrophils (**Figure 4C**, upper panel) (identified as PE-MPO and DAPI-stained multilobulated nuclei double-positive cells), which increased after Ags challenge (**Figure 4C**, lower panel). A dotted ECP signal was observed colocalizing with MPO, suggesting that ECP may be stored in azurophilic granules.

## Discussion

ECP is an allergic inflammatory mediator that has been attributed exclusively to eosinophils (2). However, no IgE-dependent production of ECP or other inflammatory mediators has been detected to date in these cells (16, 49). Previous work described ECP in neutrophils (7, 8, 16), the most abundant leukocytes and the first reaching the allergic inflammatory foci (9). We reported previously that neutrophils synthesize *de novo*, and release ECP in response to Ags/α-IgE IgE-receptor crosslinking (16), suggesting their contribution to allergic inflammation.

**Figure 5:**
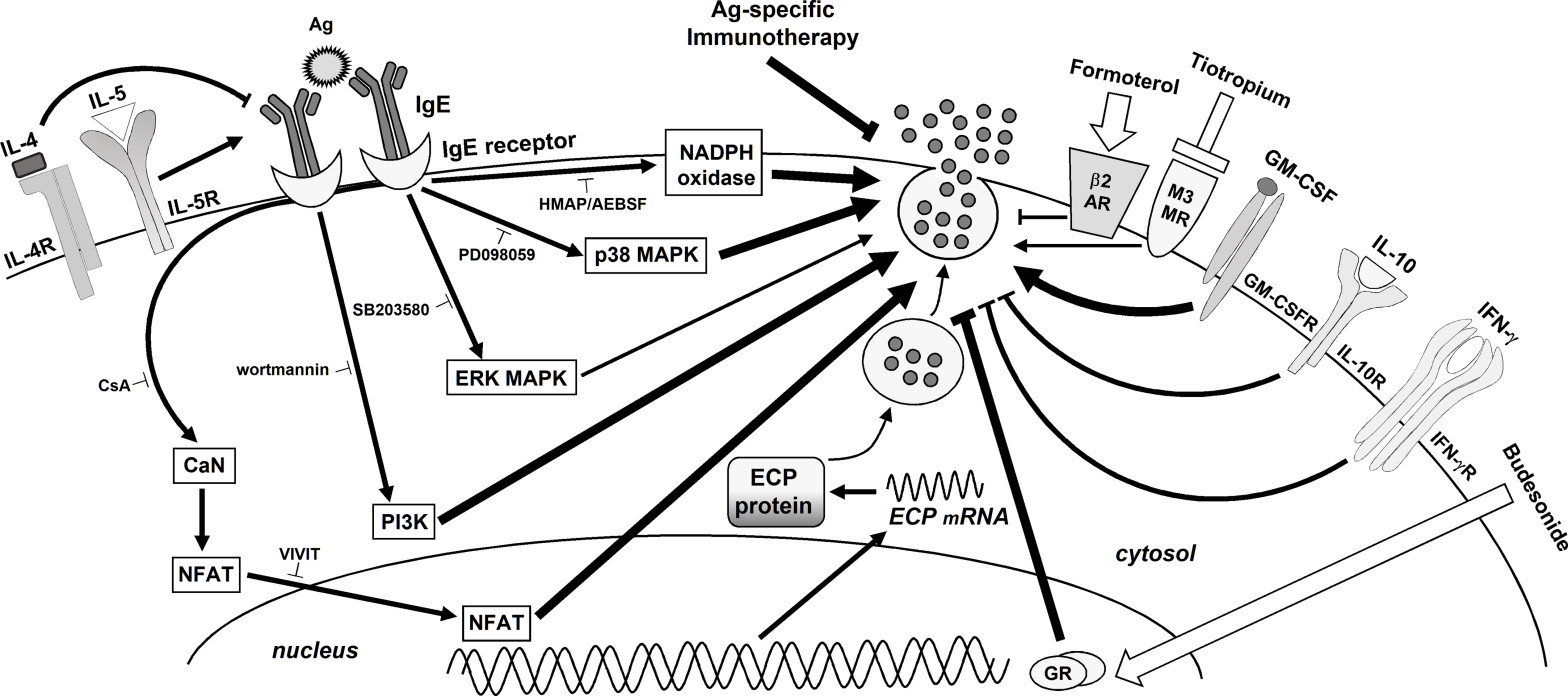
Schematic summary of the signaling pathways involved in Ags-induced ECP release by human neutrophils. Modulation by cytokines, therapeutic medical drugs and Ags-specific IT. The binding of an Ag to the specific IgE/IgE receptors complex on the neutrophil surface initiates a cascade of events activating PI3K, MAPKs (ERK and p38 and) NADPH oxidase and CN/NFAT, which leads to ECP release. The modulating effects of cytokines, glucocorticoids, LABAs, LAMAs, and Ags-specific IT are also shown. Note that the thickness of the arrows/suppressing lines represents the relative contribution of each pathway, cytokine or medical approach to ECP release.

In this work we show the cell-signaling pathways involved in ECP production, in order to provide possible therapeutic targets. We found that PI3K and ERK1/2 MAPKS are activated in response to Ags (see **Figure 5**), matching previous results from our laboratory (24, 25). They are both involved in the IgE-dependent L-selectin downmodulation (50), NF-κB activation, COX-2 expression (25), MMP-9 (51) and histamine release (24). In agreement with these results, PI3K and ERK1/2 MAPK are also required for Toll-like receptor-dependent ECP production by human eosinophils (52). In turn, eotaxin-induced eosinophil ECP release is dependent on ERK 1/2 MAPK but also on p38MAPK (53) (which is not required in neutrophils). We also show evidence of ROS involvement through NAPDH oxidase and of the calcineurin/NFAT pathway (**Figure 5**). Similarly, the antioxidant taurine-chloramine prevents fMLP-triggered NADPH oxidase activation/ROS generation and ECP production by human eosinophils (54). In these cells, CsA also inhibits the serum-coated Sephadex beads/IL-5-dependent ECP release (55), mimicking the effect that we found in neutrophils.

Allergic asthmatic inflammation is regulated by a complex network of mutually interacting soluble mediators. For instance, the T_H_2 cytokines IL-4 and IL-5 (34) and GM-CSF (35) are critical for the pathophysiology of the disease. Others, such as the T_H_1 cytokine IFN-γ (36) and IL-10 (37) are negative regulators. Cytokines are mostly ineffective or have a weak effect on ECP production by eosinophils. However, some enhance other stimuli, for instance complement components (56). In the search of other modulators, we studied the effect of cytokines on ECP production by neutrophils. Whereas IL-4 treatment had no effect on ECP release, matching data published in eosinophils (57), GM-CSF not only enhanced the action of Ags/α-IgE, as described for other agonists (56), but also stimulated *per se*. IL-5 also performed an enhancing action. Interestingly, neither the stimulating effect of GM-CSF, nor the enhancing effect of IL-5, have been reported in eosinophils (56). In turn, IFN-γ and IL-10 produced a strong inhibitory effect. These are the first insights of these immune regulators on ECP production. As an attempt to understand whether the inhibition of ECP produced by neutrophils could be of therapeutic interest, we tested the effect of current anti-allergic treatments. Glucocorticoids inhibit the endogenous generation of proinflammatory mediators while enhancing anti-inflammatory mediators, whereas LABAs/LAMAs prevent bronchoconstriction of the airways. In human neutrophils, the expression of glucocorticoid receptors (58) controls neutrophil activation, migration, respiratory burst, and apoptosis (59). In addition, they regulate IgE-dependent histamine production by neutrophils from allergic patients (24). Here we show that budesonide inhibits Ags/α-IgE-dependent ECP production. Other studies using hydrocortisone have shown similar effects on serum-coated Sephadex beads-promoted eosinophil ECP production (60). β_2_-adrenergic (activated by LABAs) and M3-muscarinic receptors (inactivated by LAMAs) are also expressed by neutrophils (61, 62). Signaling through these receptors reduces inflammation through the inhibition of cytokine production, chemotaxis (63, 64), and histamine release (24). As with eosinophils, we found a partial inhibition with the LABA formoterol (65). However, when combined with budesonide, a synergistic effect was observed matching previous studies showing that ECP levels decrease in the sputum of asthmatic patients. This leads to an improvement of the lung function and symptom scores (66, 67). We also tested the effect of tiotropium (a LAMA) on ECP production by neutrophils. Tiotropium induces a partial inhibition and enhances the hampering effect of LABAs and glucocorticoids when combined.

IT slows allergic diseases progression providing long-term clinical benefits (42). The molecular mechanisms underlying its effects are not fully understood. Previous work from our laboratory showed that IT inhibits NF-κB activation, IL-8/TXA2 production (68), myeloperoxidase (MPO) and histamine release (24, 69, 70), respiratory burst (71), and L-selectin shedding (50). Here we show that the neutrophil ECP release, in response to Ags/α-IgE and GM-CSF, represents a novel biomarker for IT effectiveness. Our results are consistent with previous reports showing ECP levels decrease in nasal secretions and sputum from IT-treated allergic patients (43, 44).

Several phenomena may explain the lower response of neutrophils from IT-treated patients: TH_2_ inflammation, which increases in the allergen season, upregulates the expression of IgE receptors in neutrophils from allergic asthmatic patients (72); IT decreases T_H_2 inflammation (73). This IT-mediated TH_2_ reduction may subsequently decrease IgE receptor expression, reducing the neutrophil transitory inflammatory phenotype and cellular responsiveness. On the other hand, IT induces an increase in circulating immunosuppressive cytokines IFN-γ and IL-10 (74). Here we found that both cytokines inhibited *in vitro* Ags/α-IgE-, IL-5- and GM-CSF-dependent ECP release, indicating that the inhibitory effect of IT might be enhanced through IFN-γ and IL-10 production. The lower response observed in healthy donors may also be due to lower IgE receptor expression, as reported previously (72).

Finally, we evaluated the correlation between ECP levels and lung function. High sputum ECP levels are correlated with airway obstruction (46), an indirect marker of eosinophilic inflammation (2, 5). However, eosinophil counts in sputum do not always match with ECP levels (75), suggesting other sources of ECP production. Our results match previous results showing that sputum ECP levels and FEV1are inversely correlated (46). In addition, we provide new evidence showing that FEV1 inversely correlates with the *in vitro* ECP released by peripheral blood neutrophils after Ags/α-IgE challenge, a observation consistent with previous work (76).

Although ECP has been detected in sputum neutrophils from asthmatic patients (6), no functional analysis has been performed on these cells before. Here we show that Ags induce ECP expression in neutrophils from cultured sputum, indicating that these cells are an ECP source in the airways of allergic asthmatic patients that has been dismissed to date.

Our work presents neutrophil ECP production *in vitro* as a predictive marker of allergic asthma severity. The molecular pathways that we describe represent potential therapeutic targets that need to be taken into consideration for future approaches.

## Data availability statement

The raw data supporting the conclusions of this article will be made available by the authors, without undue reservation.

## Ethics statement

The Hospital Universitario Virgen Macarena ethics committee approved the study and each sample donor gave written informed consent. The patients/participants provided their written informed consent to participate in this study.

## Author contributions

AV-R and PC designed and performed most of the research, analyzed the data, and helped with the preparation of the manuscript. LF-D, LD-C and BP-M participated in the selection and recruitment of patients. BD performed some research. AR performed some research and wrote the manuscript. DR and RP analyzed the data and wrote the manuscript. JP and JR performed statistical analysis of the data and language editing. JM participated in the diagnosis of the patients, designed the research, analyzed the data, and wrote the manuscript. DR-P: analyzed the data and wrote the manuscript. All authors contributed to the article and approved the submitted version.

## Funding

This work was cofunded by the European Union through the European Regional Development Fund (ERDF) and was supported by grants from the Instituto de Salud Salud Carlos III (FIS-Thematic Networks and Co-Operative Research Centres ARADYAL, RD16/0006/0035) and Fundació n Alergol, Spain. AV-R was supported by a grant from the Ministerio de Economía y Competitividad (Proyectos I+D+i para Jóvenes Investigadores, SAF2014-60649-JIN) and holds a Nicolás Monardes contract from the Andalusian Health Service (C-0060-2018); PC is under a senior postdoc contract from the Ministry of Health and Families (Junta de Andalucía, Ref RH-0129-2020). The funders had no role in study design, data collection and analysis, decision to publish, or preparation of the manuscript.

## Acknowledgments

We would like to thank Katherina García and Jose Maria Urbano for their confocal microscopy assistance.

## Conflict of interest

RP and DR were employed by Diater.

The remaining authors declare that the research was conducted in the absence of any commercial or financial relationships that could be construed as a potential conflict of interest.

## Publisher’s note

All claims expressed in this article are solely those of the authors and do not necessarily represent those of their affiliated organizations, or those of the publisher, the editors and the reviewers. Any product that may be evaluated in this article, or claim that may be made by its manufacturer, is not guaranteed or endorsed by the publisher.

## Abbreviations

ECP, eosinophil cationic protein; ERK, extracellular signal-regulated kinase; FEV1, forced expiratory volume in first second; IL, interleukin; MAP, mitogen activated protein kinases; PI3K, phosphatidylinositol 3-kinase.
